# Neighborhood Deprivation and Treatment Challenges in Pediatric Musculoskeletal Infections: A Socioeconomic Analysis

**DOI:** 10.7759/cureus.61998

**Published:** 2024-06-09

**Authors:** Sonal Mahindroo, Shruthi Mohan, Sarah Dance, Alana O'Mara, Ahmed Elabd, Sean Tabaie

**Affiliations:** 1 Orthopaedic Surgery, George Washington University School of Medicine and Health Sciences, Washington DC, USA; 2 Orthopaedic Surgery, Children’s National Hospital, Washington DC, USA; 3 Orthopaedic Surgery, Children's National Hospital, Washington DC, USA

**Keywords:** socioeconomic status (ses), septic arthritis, osteomyelitis, area deprivation index (adi), pediatric orthopedic surgery, musculoskeletal infection

## Abstract

Introduction

Musculoskeletal (MSK) infections are prevalent in the pediatric population, with previous research highlighting the significant impact of socioeconomic status (SES) on treatment outcomes. However, the specific link in pediatric cohorts remains poorly understood. The Area Deprivation Index (ADI), a measure of neighborhood-level disadvantage, serves as a crucial marker for SES. This study aims to investigate how ADI influences disease characteristics, treatment delays, and outcomes in pediatric patients with MSK infections.

Methods

A single-center retrospective cohort analysis was conducted using patient charts from a large urban pediatric hospital over six years from 2017 to 2022. Patients aged 0-18 years with diagnoses of osteomyelitis, septic arthritis, cellulitis, or pyomyositis were identified using the International Classification of Diseases - 10th Revision (ICD-10) codes. Data collection included demographics, disease characteristics, treatment delay intervals, and complications. Patient zip codes were obtained and entered into the Neighborhood Atlas® mapping website to determine their ADI. Patients were then stratified into four groups based on ADI scores: 1-10, 11-20, 21-40, and 41-100. Statistical analysis included the use of the Mann-Whitney U test for continuous data and the Chi-square/Fisher’s exact test for binary and categorical data comparisons among the ADI groups.

Results

A total of 121 patients were included. Categorization based on ADI revealed 25 (20.7%) patients in the 1-10 ADI percentile group, 36 (29.8%) in the 11-20 group, 38 (31.4%) in the 21-40 group, and 22 (18.2%) in the 41-100 group. There were no significant differences between ADI and patient demographics, disease characteristics, presentation delay interval, treatment received, and complications.

Conclusion

The study demonstrates that there was no significant difference between ADI groups regarding demographics, disease characteristics, presentation delay interval, treatment received, and complications in pediatric populations. Despite the lack of evidence for differences in MSK infections attributable to ADI, this does not negate the potential existence of such a relationship.

## Introduction

Musculoskeletal (MSK) infections, such as osteomyelitis, septic arthritis, and pyomyositis, are increasingly prevalent among pediatric populations, affecting approximately 80 out of 100,000 children annually [1]. *Staphylococcus aureus* remains the most common causative organism, isolated in about 40% to 90% of cases of MSK infection, although other pathogens like *Kingella kingae* are also emerging as notable pathogens, particularly in younger children [2-4] Without timely treatment, these infections can lead to severe complications such as chronic infections, bacteremia, and sepsis, emphasizing the critical need for prompt diagnosis and appropriate treatment [5].

In the realm of pediatric healthcare, disparities based on patients' demographics and socioeconomic factors are well-documented [6]. Lower socioeconomic status (SES) has been linked to delays in accessing MSK care, a concern especially relevant in pediatric orthopedics [7]. Studies have highlighted how delayed surgery and residence in rural areas can lead to adverse outcomes, particularly in cases of osteomyelitis [8,9]. While previous research has explored disparities in general MSK disorders, there remains a gap in understanding their specific impact on the characteristics and treatment outcomes of MSK infections in pediatric patients.

The Area Deprivation Index (ADI) is a scoring system derived from residential addresses that consider multiple socioeconomic factors, such as income, education, employment, and housing quality, within a specific geographical area [10]. This index serves as a proxy measure for assessing the prevailing socioeconomic conditions in each neighborhood by zip code and is, therefore, employed in this study to gauge the SES across different neighborhoods.

The purpose of the study is to investigate the potential link between ADI and the characteristics of MSK infections in pediatric populations. Additionally, the present study aims to assess any delays in treatment initiation and examine the impact of SES on treatment outcomes.

## Materials and methods

Study design

The present study was a retrospective chart review of pediatric patients (age 0 to 18) diagnosed with MSK infections at a single, tertiary pediatric care facility within a period spanning from 2017 to 2022. International Classification of Diseases - 10th Revision (ICD-10) codes were used to identify patients diagnosed with osteomyelitis, septic arthritis, cellulitis, or pyomyositis. Patients who were not definitively treated at the study institution, were treated by non-orthopedic surgeons, or did not have a zip code available in their chart were excluded. All patients from the study period who met inclusion and exclusion criteria were included. The study was determined to be exempt from review by the local institutional review board (IRB).

Data collection

Each patient’s zip code was obtained from their chart and inputted into the Neighborhood Atlas® mapping website to determine the ADI [11]. A higher ADI number indicates that the patient lives in a more disadvantaged neighborhood with a higher level of deprivation, while a lower ADI suggests that the patient lives in a more advantaged area with a lower level of deprivation [12]. Subsequently, patients were categorized into four distinct groups according to their ADI percentiles: Group 1 (1-10th percentile), Group 2 (11-20th percentile), Group 3 (21-40th percentile), and Group 4 (41-100th percentile).

For each included patient, the following data were collected: demographics (age and gender), disease characteristics (type of infection, type of culture, and culture results), presentation time interval (days from onset of symptoms to date of presentation to the emergency department (ED)), treatment received (surgical or non-surgical), and complications (defined as readmission and/or repeat surgery) within 30 days of initial discharge.

Statistical analysis

Patient demographics, disease characteristics, presentation time interval, treatment received, and disease outcome were compared among the four ADI groups using the Mann-Whitney U test for statistical analysis of continuous data and Chi-square/Fisher’s exact test for binary and categorical data. P-values were obtained, with a value less than 0.05 denoting statistical significance.

## Results

Demographics and patient categorization

**Figure 1:**
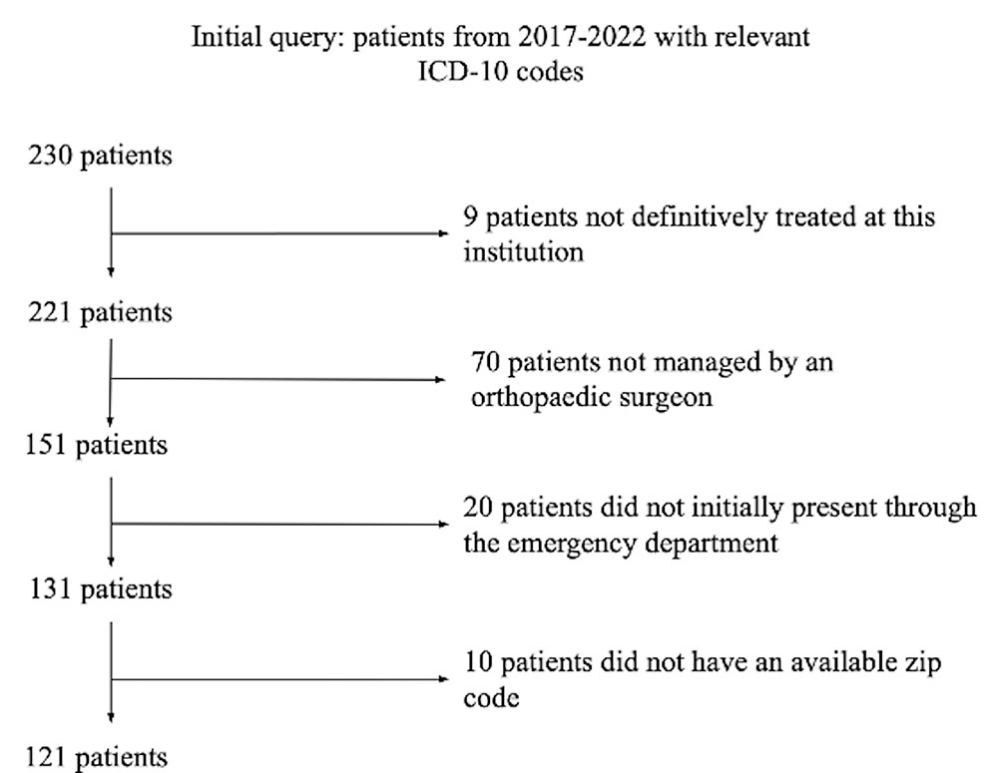
Inclusion and Exclusion Criteria for Patient Cohort

Out of the 230 patients initially identified, 109 patients were excluded, yielding a total of 121 patients included in the analysis. A summary of the selection process is shown in Figure 1. The median age of the included subjects was seven years (range 0.1-18 years). Categorizing patients in four ADI groups showed 25 patients (20.7%) in the 1-10th ADI percentile group, 36 (29.8%) in the 11-20th, 38 (31.4%) in the 21-40th, and 22 (18.2%) in the 41-100th group. Additional data pertaining to demographics and patient categorization can be found in Table 1. There was no statistical difference between patients in the four ADI groups pertaining to demographics.

**Table 1:** Summary Demographics of Patient Cohort ADI, Area Deprivation Index

Variables	Overall (n=121)
Age (years), median (range)	7.0 (0.1, 18.0)
Gender (Male:female)	78:43
ADI category, n (%)	
1-10th percentile	25 (20.7)
11-20th percentile	36 (29.8)
21-40th percentile	38 (31.4)
41-100th percentile	22 (18.2)
ADI, median (range)	20.0 (1.0, 76.0)
Infection type, n (%)	
Osteomyelitis	70 (57.9)
Septic arthritis	35 (28.9)
Cellulitis	10 (8.3)
Pyomyositis	6 (5.0)
Type of positive culture, n (%)	
Wound	46 (44.2)
Blood	51 (49.0)
Blood and wound	7 (6.7)
Organisms isolated, n (%)	
Staphylococcus aureus	52 (50.0)
Streptococcus pyogenes	13 (12.5)
Other	8 (7.7)
Negative	31 (29.8)
Days from symptoms to presentation, median (Range)	4 (0.0, 180.0)
Surgery, n (%)	93 (76.9)
30-day complication rate, n (%)	11 (9.1)
ADI, median (range)	20.0 (1.0, 76.0)

Disease characteristics

The most common pediatric MSK infection was osteomyelitis (57.9%), followed by septic arthritis (28.9%), cellulitis (8.3%), and pyomyositis (5.0%). There was no difference in the type of infection reported between the four ADI groups.

Out of 121 patients, 104 (86%) had a positive culture (either surgical or blood or both) prior to treatment initiation. Of these 104 patients who had positive cultures, 46 (44%) patients had positive surgical cultures only, 51 (49%) had positive blood cultures only, and seven (7%) patients had positive surgical and blood cultures. Comparing types of positive cultures between the four ADI groups showed no difference with the blood culture type (versus surgical culture type) being the most likely to be positive in all groups.

In terms of organisms isolated in a positive culture, *Staphylococcus aureus* was the most common infectious organism in this patient cohort (50.0%) followed by Streptococcus *pyogenes *(12.5%). Comparing the organism isolated from positive cultures between the four ADI groups showed no difference with *Staphylococcus aureus* being the most common organism isolated in all ADI groups.

Presentation delay and treatment received

The median number of days from symptom onset to presentation to the ED was four days (0-180). Similarly, there was no difference in time interval between the four ADI groups. Most patients in the cohort (76.9%) received surgical treatment as opposed to medical management alone with no difference between the four ADI groups.

Outcomes and complications

The incidence of 30-day complications (defined as readmission to the ED and/or repeat surgery) in this cohort was 11% with no difference between the four groups as seen in Table 2.

**Table 2:** Correlation of MSK Infection Characteristics by ADI Group A p-value <0.05 was considered statistically significant. - denotes p-value not available. ADI, Area Deprivation Index; MSK, musculoskeletal

Outcomes	ADI 1-10th (n=25)	ADI 11-20th (n=36)	ADI 21-40th (n=38)	ADI 41-100th (n=22)	P-value
Infection, n (%)					-
Osteomyelitis	14 (56.0)	21 (58.3)	23 (60.5)	12 (54.5)	0.969
Septic arthritis	7 (28.0)	12 (33.3)	11 (28.9)	5 (22.7)	0.859
Cellulitis	3 (12.0)	1 (2.8)	4 (10.5)	2 (9.1)	0.539
Pyomyositis	1 (4.0)	2 (5.6)	0 (0.0)	3 (13.6)	0.134
Type of culture, n (%)					0.713
Blood	11 (45.8)	17 (60.7)	13 (38.2)	10 (55.6)	-
Wound	11 (45.8)	10 (35.7)	18 (52.9)	7 (38.9)	-
Blood and wound	2 (8.3)	1 (3.6)	3 (8.8)	1 (5.6)	-
Organism isolated from culture, n (%)					0.446
Staphylococcus aureus	8 (33.3)	16 (57.1)	20 (58.8)	8 (44.4)	0.705
Streptococcus pyogenes	5 (20.8)	2 (7.1)	3 (8.8)	3 (16.7)	-
Other	2 (8.3)	3 (10.7)	2 (5.9)	1 (5.6)	-
Negative	9 (37.5)	7 (25.0)	9 (26.5)	6 (33.3)	-
Days from symptom onset to presentation, median (Range)	8 (0.0, 57.0)	8 (1.0, 38.0)	7 (1.0, 49.0)	13 (0.0, 180.0)	0.550
Surgery, n (%)	5 (20.0)	14 (38.9)	5 (13.2)	4 (18.2)	0.055
30-day complication rate, n (%)	4 (16.0)	3 (8.3)	2 (5.3)	2 (9.1)	0.543

## Discussion

As previous studies have highlighted SES as influencing MSK outcomes in the pediatric population, the present study sought to determine any correlation of ADI with MSK infections. After analyzing pediatric patients being treated for MSK infections at our single institution, there were no statistically significant differences in patient demographics, disease characteristics, time to presentation, treatment received, and complications based on patient SES, as defined by ADI. It is worth noting that the study was conducted in a large metropolitan city, with only 18% of the study cohort belonging to the 41-100th percentile ADI group, representing the highest deprivation index. This lower representation in the highest ADI group may have contributed to the study's inability to detect significant differences among the four studied groups.

The present study found that osteomyelitis was the most prevalent pediatric MSK infection, consistent with prior research indicating a higher incidence of osteomyelitis compared to cellulitis and septic arthritis in this population [5,13,14]. Additionally, *Staphylococcus aureus* was identified as the primary causative organism, accounting for 82.8% of cases. This is also in line with established literature trends; however, *Streptococcus pyogenes* was also implicated in a minority of our cases.

Our study also examined the role of culture types, such as blood or wound cultures, in identifying common pathogens across different ADI percentile groups. While we did not observe significant differences in pathogen identification based on culture type and ADI percentiles, it is noteworthy that most patients underwent either blood or wound cultures rather than both. Emphasizing the significance of culture types, particularly blood cultures, is crucial for the initial assessments of MSK infections in pediatric populations. This approach facilitates early recognition of specific targets in antibiotic management, highlighting the importance of a comprehensive diagnostic strategy in pediatric MSK infections [15].

While Popescu et al. reported that children from rural areas with higher trauma exposure had limited access to urgent medical care, resulting in delayed hospital presentations and poorer outcomes in acute hematogenous osteomyelitis, our study did not find a significant difference between deprivation index levels and the time from symptom onset to presentation at our ED [8]. The dissonance observed between the two studies may be attributed to Popescu et al. determining SES solely through clinical chart review. Whittington et al., on the other hand, used the ADI measure in rural pediatric populations, focusing on *Staphylococcus aureus* bacteremia due to osteomyelitis and other systemic infections [16]. They also found no association between ADI and treatment failure, measured by mortality and readmission rates. Similarly, the current study found no significant relationship between ADI and 30-day complication rates, measured by readmission to the emergency room and the need for repeat surgery. Unfortunately, our study lacked sufficient data to assess compliance with follow-up or outpatient treatment plans. This aspect should be a focus in future studies due to the potential impact of neighborhood deprivation on access to transportation, healthcare facilities, and health education, all of which can affect timely symptom recognition and management [17].

While the present study did not find a clear relationship between SES (represented by ADI) and various disease characteristics and treatment outcomes, the lack of evidence does not negate the potential existence of such a relationship. Rather, it emphasizes the need for additional studies that gather more detailed data reflecting the comprehensive care and clinical trajectory of MSK infections. By gaining more specific insights, orthopedic physicians can better understand and address these barriers, providing individualized care that goes beyond what we were able to elucidate in our study. Therefore, ongoing efforts to explore the effects of SES on pediatric care will contribute significantly to identifying areas for improvement, particularly for at-risk patient populations, and more broadly, for enhancing our healthcare system.

The present study has several limitations that should be discussed. First, being a cohort study, there is a potential for selection and observer's bias inherent to the study design. Additionally, the findings may not be readily generalizable as they are based on data from a single institution, limiting the broader applicability of the results. Moreover, while the ADI is a validated measure of socioeconomic disadvantage, its use can sometimes reflect the median value of neighborhood homes rather than the actual level of disadvantage, particularly in regions with wide variations in the cost of living. This could potentially skew the study results, especially in areas where housing prices are high but disadvantaged communities still exist [18]. Furthermore, the inclusion of pyomyositis, although not typically managed by orthopedic surgeons, was done due to its association with MSK infectious pathologies as indicated in the literature [5,13]. However, it is important to note that the management of these pathologies often involves a multi-specialty approach, whereas this study focused solely on orthopedic management [19]. Future research should explore the relationship between SES and outcomes after the multidisciplinary management of MSK infections.

## Conclusions

The present study demonstrated that there were minimal differences between ADI groups regarding demographics, disease characteristics, presentation delay interval, treatment received, and complications in a pediatric population. Despite the lack of evidence for differences in MSK infections attributable to ADI, this does not negate the potential existence of such a relationship. Rather, the present study emphasizes that additional studies evaluating SES and MSK infection outcomes are necessary to better guide orthopedic surgeon management decisions.
